# Customer‐Oriented Development and Characterization of Spice‐Enriched Extruded Breakfast Cereal From Rice Bran and Unripe Banana Flour

**DOI:** 10.1111/1750-3841.71475

**Published:** 2026-09-16

**Authors:** Zaki Muhammad Husyemi Rafsanjani, Slamet Budijanto, Vallerina Armetha, Azis Boing Sitanggang

**Affiliations:** ^1^ Division of Food Science and Technology, Faculty of Engineering and Technology, IPB Dramaga Campus IPB University Bogor Indonesia; ^2^ Division of Chemical Engineering, Design, and Smart Manufacture, Faculty of Engineering and Technology IPB Dramaga Campus IPB University Bogor Indonesia; ^3^ Graduate School of Agricultural Science Tohoku University Miyagi Japan

## Abstract

Developing functional breakfast cereals that are both nutritionally enhanced and consumer‐acceptable remains a key challenge for product innovation. This study developed a consumer‐oriented, spice‐enriched extruded breakfast cereal formulated by partially substituting corn grits with rice bran and unripe banana flour, with product optimization guided primarily by sensory evaluation. Four formulations containing 0%–3% of a spice blend (ginger, cinnamon, and lemongrass) were produced using extrusion cooking and evaluated under three serving conditions (without milk, with cold milk, and with warm milk). Product development followed a three‐step approach. First, sensory differences among formulations were assessed using Check‐All‐That‐Apply (CATA), Rate‐All‐That‐Apply (RATA), and hedonic rating tests, revealing significant variations in aroma, taste, and aftertaste attributes. Across serving conditions, the formulation containing 1% spice achieved the highest overall liking (*p* < 0.001 without milk; *p* < 0.05 with warm milk). Functional characterization showed that increasing spice levels significantly enhanced total phenolic content (17.53–29.16 mg GAE/g), total flavonoid content (9.51–26.96 mg QE/g), and antioxidant activity (6.08–103.36 mg TE/g ABTS; 4.25–30.74 mg TE/g DPPH) (*p* < 0.001). Second, the optimal formulation was identified using Bayesian multi‐criteria decision analysis integrating sensory acceptance with nine physicochemical parameters, confirming the 1% spice blend (SB) as optimal. Third, the selected formulation was further characterized for glycemic response. The glycemic index of the optimized product was 47 ± 8, categorizing it as a low‐glycemic food. These findings demonstrate that modest spice incorporation can maintain consumer preference while improving functional properties, supporting the development of healthier extruded cereal products.

**Practical Applications:**

This study provides a practical framework for developing spice‐enriched extruded breakfast cereal (SEBC) using rice bran and unripe banana flour while maintaining consumer acceptance. The consumer‐oriented product development approach enables food manufacturers to optimize spice levels to achieve a desirable balance between flavor, texture, and functional properties without compromising consumer acceptance. The study further demonstrates the feasibility of converting underutilized agricultural by‐products (i.e., rice bran and unripe banana flour) into commercially viable, functional cereal products with improved nutritional attributes.

## 1 Introduction

The global burden of non‐communicable diseases (NCDs), including diabetes, cardiovascular diseases, and obesity, continues to increase at an alarming rate. In 2024 alone, diabetes affected approximately 589 million adults (11.1% of the global population, resulting in over 3.4 million deaths and nearly $1 trillion in healthcare expenditure (International Diabetes Federation 2026). These trends highlight the urgent need for dietary strategies that extend beyond basic nutrition, particularly as unhealthy diets, obesity, and physical inactivity remain major modifiable risk factors. At the same time, global food security challenges persist, with nearly one billion individuals unable to meet their nutritional needs. This dual burden underscores the importance of developing functional foods that are not only nutritionally enhanced but also accessible and acceptable, especially in developing regions (Ahrné et al. 2025).

In response, the food industry has increasingly focused on designing products that combine convenience with health‐promoting properties, such as functional attributes that support long‐term health. Consequently, attention has shifted toward commonly consumed food categories that meet the demands of modern lifestyles, with breakfast cereals emerging as a key vehicle due to their high consumption frequency and adaptability for functional formulation. Extrusion technology has emerged as a highly efficient and scalable method for producing a wide range of cereal‐based products. This process operates under rapid thermomechanical conditions (114.5°C–170.0°C, 20–40 s), enabling continuous production with high throughput and consistent quality while maintaining energy efficiency (Mohammed et al. 2024; Boakye et al. 2023; S. S. Lim et al. 2016). During extrusion, key physicochemical transformations occur, including starch gelatinization, protein denaturation, enzyme inactivation, and microbial reduction. Despite these advantages, conventional extruded cereals often lack sufficient functional attributes to address current health concerns.

Functional foods, that are defined as foods that provide health benefits beyond basic nutrition, have gained increasing attention as potential dietary interventions for NCDs prevention. Breakfast cereals are particularly suitable vehicles for functional ingredient delivery due to their widespread consumption and formulation flexibility. Enrichment with dietary fiber, polyphenols, and other bioactive compounds has been shown to mitigate oxidative stress and chronic inflammation, which are key mechanisms underlying degenerative diseases (Ma et al. 2025). In this context, spices and herbs represent cost‐effective sources of bioactive compounds. Beyond enhancing sensory attributes, spices such as ginger, cinnamon, and lemongrass exhibit strong antioxidant and antimicrobial properties, contributing both to product stability and potential health benefits (Shah et al. 2011; Verma et al. 2025; Hamidpour et al. 2015).

Rice bran and unripe banana flour are promising functional ingredients for cereal‐based applications. Rice bran is rich in bioactives, including tocotrienols, γ‐oryzanol, and dietary fibers such as beta‐glucan (Rafe et al. 2017). Unripe banana flour provides resistant starch, which supports gut health and contributes to a lower glycemic response, making it particularly relevant for the management of type 2 diabetes risk (Gamlath 2008). The incorporation of spices further enhances functional potential through additional polyphenols and flavonoids, while also introducing distinctive aromatic profiles. Notably, the combination of ginger, cinnamon, and lemongrass draws from their traditional use in herbal beverages, offering a familiar sensory profile that may enhance consumer acceptance across different serving conditions (Zhang et al. 2024; Francis et al. 2005).

Previous works by Sukarno et al. (2022) demonstrated the feasibility of producing extruded cereals from rice bran and banana flour with acceptable sensory properties. However, a significant reduction in antioxidant activity was observed following extrusion, highlighting a critical limitation in preserving bioactives under thermomechanical processing. To date, the combined use of rice bran, unripe banana flour, and selected spices as complementary sources of antioxidants in extruded breakfast cereals has not been systematically investigated. This gap suggests an opportunity to develop formulations that simultaneously enhance functional properties and maintain consumer acceptability.

This study aimed to investigate the effects of varying concentrations of a blended spice powder (ginger, cinnamon, and lemongrass) in extruded breakfast cereals formulated from rice bran and unripe banana flour on their physical, functional, and sensory characteristics. While previous studies have explored rice bran–banana flour cereals, the integration of spice‐derived bioactive compounds within this matrix, combined with a consumer‐oriented formulation strategy, remains limited. By incorporating sensory‐driven optimization alongside physicochemical and glycemic evaluation, this work provides a comprehensive approach to developing extruded cereals that balance functional enhancement with consumer acceptability. Furthermore, this study contributes to the valorization of local agricultural resources and supports the development of functional foods that may help mitigate diet‐related degenerative diseases.

## 2 Materials and Methods

### 2.1 Human Subject Assurance

This study was conducted in accordance with ethical standards and was approved by the Research Ethics Committee of IPB University (approval no. 1891/IT3.KEPMSM‐IPB/SK/2025). Written informed consent was obtained from all participants prior to their involvement in the study. All sensory evaluations and glycemic response measurements were performed only after participants had completed the consent process.

### 2.2 Materials

Corn starch was obtained from Ega Multi Cipta Co. Ltd. (Jakarta, Indonesia). Rice bran derived from Inpari 32 was provided by Gapoktan Sidomulyo (Sleman, Indonesia). Unripe banana flour was sourced from KWT Melati (Kulon Progo, Indonesia). Ginger and cinnamon powders were purchased from Gunacipta Multirasa Co. Ltd. (Tangerang, Indonesia), while lemongrass powder was obtained from the Spices Industry (Indonesia). Skimmed milk powder, palm sugar, refined salt, and fresh milk were purchased from a local market. A commercial breakfast cereal produced by a multinational manufacturer (Buffalo, New York, USA) was used as the reference sample.

Analytical grade reagents, including DPPH (2,2‐diphenyl‐1‐picrylhydrazyl), Trolox (6‐hydroxy‐2,5,7,8‐tetramethylchroman‐2‐carboxylic acid), methanol, ABTS (2,2′‐azino‐bis(3‐ethylbenzothiazoline‐6‐sulfonic acid)), gallic acid, quercetin, aluminum chloride (AlCl_3_), phosphate buffer (0.08 M, pH 6.0) were obtained from Sigma‐Aldrich (St. Louis, MO, USA). Enzymes used for in vitro digestion included heat‐stable α‑amylase and protease from *Bacillus licheniformis*, and amyloglucosidase from *Aspergillus niger* (Sigma‐Aldrich, St. Louis, MO, USA). Ethanol (absolute, 95%, and 78% v/v), Folin–Ciocalteu reagent, sodium carbonate (Na_2_CO_3_), potassium persulfate (K_2_S_2_O_8_), sodium hydroxide (NaOH), hydrochloric acid (HCl), and acetone were of analytical grade and purchased from Merck (Darmstadt, Germany). Celite 545 was obtained from Macklin (Shanghai, China). Whatman No. 42 ashless filter paper was used for dietary fiber analysis. Dextrose monohydrate (Brataco Chemicals, Indonesia) was used as the reference food for glycemic index (GI) measurement.

Blood glucose levels were measured using an Accu‐Chek Active glucometer (Roche, Basel, Switzerland) with corresponding glucose test strips and lancets (Accu‐Chek Softclix, Roche, Basel, Switzerland). Blood sampling was performed using 70% alcohol wipes (Arista Latindo, Indonesia).

### 2.3 Rice Bran Preparation

Rice bran was obtained by twice milling paddy rice using a Yanmar Huller (HW‐60 A, Osaka, Japan) for approximately ±10 min followed by twice polishing with a Satake Rice unit (N‐70 F, Tokyo, Japan) for approximately ±40 s. The bran was stabilized using a twin‐screw extruder (Customized Berto Co., Indonesia) at 120°C and 15 Hz without a die to minimize oxidation during storage.

### 2.4 Production of Spiced‐Enriched Extruded Breakfast Cereal (SEBC)

Extruded cereal was produced following previously reported methods (Budijanto et al. 2012; Sukarno et al. 2022). Formulations were prepared on a 2 kg batch basis with varying concentrations of spices blend (Table 1). Dry ingredients (corn starch, stabilized rice bran, unripe banana flour, skimmed milk powder, palm sugar, and spice powder) were dry‐blended in a blade mixer (Sigma, Indonesia) at medium speed for 5 min. Salt was dissolved in water and added to the mixture, followed by wet blending for an additional 5 min to obtain a homogeneous dough. The dough was subsequently processed using a twin‐screw extruder operated at a screw speed of 40 Hz and a cutter speed of 50 Hz. The extrudates were cooled to ambient temperature and packaged in polypropylene (PP) zip‐lock bags prior to analysis.

**TABLE 1:** Spice‐enriched extruded breakfast cereal formulations.

Raw material	Formula			
	*F*0	*F*1	*F*2	*F*3
Corn starch (g)	1100	1100	1100	1100
Rice bran (g)	400	400	400	400
Banana flour (g)	200	200	200	200
Salt (g)	20	20	20	20
Water (mL)	105	105	105	105
Skimmed milk (g)	100	100	100	100
Palm sugar (g)	60	60	60	60
Ginger (g)	—	15	30	45
Cinnamon (g)	—	4	8	12
Lemongrass (g)	—	1	2	3

*Note: F*0 = formula with 0% SB—Control; *F*1 = formula with 1% SB; *F*2 = formula with 2% SB; *F*3 = formula with 3% SB.

Abbreviation: SB, Spices blend.

### 2.5 Experimental Design

A completely randomized design was applied using four spice‐blend levels: 0% (control) for *F*0, 1% for *F*1, 2% for *F*2, and 3% for *F*3. These concentrations were selected through preliminary trials with modification based on Amer and Rizk (2022), who used single‐spice formulations. Each formulation (Table 1) was prepared in two independent production runs; while the proportion of the base ingredients, corn starch, rice bran, banana flour, salt, water, skimmed milk, and palm sugar, were maintained across treatments. The best‐performance formula was selected using Bayesian multi‐criteria analysis, based on the overall hedonic score and selected physical attributes. The weighing criteria were assigned a priori on a 1–8 scale (worst to best), with overall hedonic liking receiving the highest priority because consumer acceptance was the principal product‐development objective. The selected formulation was subsequently analyzed for total dietary fiber (TDF) and GI.

### 2.6 Physical Characterization of Spice‐Enriched Extruded Breakfast Cereal (SEBC) Properties

#### 2.6.1 Color

Color was measured using a CR‐300 Chromameter (Konica Minolta, Japan), calibrated against a standard white plate before analysis. Five extrudate pieces were randomly selected from each production run, and measurements were taken at three random positions on each piece. The results were expressed using CIE L*a*b* color space. The total color difference (Δ*E*) was calculated to quantify the visual difference among formulations (Gupta et al. 2021).

#### 2.6.2 Cereal Resistance in Milk

Cereal resistance in milk was evaluated as indicator of crispness retention during consumption. A 1.5 g cereal sample was immersed in 70 mL of milk maintained at 18°C. Resistance time was recorded from the initial contact with milk until the cereal loss its acceptable crispness. The measurement was conducted in triplicate for each production run (Papunas et al. 2013).

#### 2.6.3 Texture Properties

Texture was measured using a TA1 texture analyzer (Lloyd Instruments AMETEK, United Kingdom) equipped with a 6‐mm cylindrical probe (SMS P/6), following a previously reported method with minor modification. Individual cereal pieces with an approximate thickness of 9 mm were compressed to 75% strain at a test speed of 1 mm/s. Hardness was expressed in Newton, while crispness/crunchiness was expressed in N.mm. Five cereal pieces were measured from each production run (Oliveira et al. 2018).

#### 2.6.4 Bulk Density

Bulk density (BD) was determined using a modified protocol based on Ezegbe et al. (2025). The cereal samples were gently poured into a 50‐mL graduated cylinder (IWAKI Asahi Glass, Japan) and tapped repeatedly until the sample volume remain constant stabilization. BD was calculated as the ratio of sample mass to volume and expressed as g/mL. Five replicate measurements were conducted for each production run.

#### 2.6.5 Water Absorption Index and Water Solubility Index

The water absorption index (WAI) and water solubility index (WSI) were determined in triplicate following Anderson et al. (1970) with minor modification. Ground samples (2.5 g, dry basis) were mixed with 30 mL distilled water, vortexed at 25°C for 30 min (DLAB MX‐S, China), and centrifuged at 3000 rpm for 15 min (Megafuge ST4 Plus, ThermoFisher, USA). For WSI, 10 mL of supernatant was dried at 105°C to constant weight and expressed as a percentage of the initial dry sample. WSI was calculated as the weight of hydrated residue per unit dry sample (Sebastião et al. 2023).

#### 2.6.6 Sectional Expansion

Sectional expansion (SE) was determined based on extrudate diameter measurements described by Senevirathna et al. (2022). Ten samples per formulation were measured using a vernier caliper (Tricle Brand, China), and the expansion index (index of SE) was calculated as the ratio of extrudate diameter to die diameter.

### 2.7 Sensory Characterization of SEBC

Sensory attributes evaluated using Check‐All‐That‐Apply (CATA), Rate‐All‐That‐Apply (RATA), and hedonic tests were based on descriptors previously identified through quantitative descriptive analysis (QDA) (Rafsanjani et al. 2026). The commercial cereal used in the preceding QDA study served only as an external market reference for descriptor development and was not treated as the experimental control in the present sensory evaluation.

A total of 70 untrained consumer panelists was selected to be participated in the study. Prior to evaluation, panelists completed an ideal‐profile task describing their preferred breakfast cereal characteristics. The sensory evaluation was conducted in a single session divided into three evaluation blocks corresponding to the serving conditions: dry cereal, cereal with cold fresh milk at 8°C, and cereal with warm fresh milk at 30°C. The three conditions were evaluated sequentially on the same day, with an approximately 1‐h interval between blocks to minimize sensory fatigue and carryover effects. Within each block, participants evaluated all four formulations.

In the CATA test, panelists selected all sensory attributes that applied to each sample. In the RATA test, the perceived intensity of each selected attribute was rated using a 5‐point Likert scale (1 = *very low intensity*; 5 = *very high intensity*) (Ares et al. 2014). Overall acceptability was assessed using a 6‐point hedonic scale ranging from 1 (*dislike very much*) to 6 (*like very much*) (Kusdiana et al. 2020). The even‐numbered scale was selected for hedonic test to remove the neutral midpoint (reduce error/bias caused by central tendency) and encourage participants to indicate a directional response toward either liking or disliking during comparative product screening. It also provided sufficiently distinct and readily understandable response categories for untrained consumers (Lawless and Heymann 2010; J. Lim 2011).

### 2.8 Proximate Analysis

Proximate composition was determined using standardized methods. Moisture, ash, and fat contents were analyzed according to SNI 01–2354:2006 (Indonesian National Standard 2006b). Protein content was determined using the Kjeldahl method following AOAC 960.52‐1961 (Association of Official Analytical Chemist 2010) and TDF was analyzed following AOAC 985.29 (Association of Official Analytical Chemist 2005). Carbohydrate content was calculated by difference as 100% minus the sum of moisture, protein, fat, and ash.

### 2.9 Functional Properties of SEBC

#### 2.9.1 Sample Extraction

Phenolic and flavonoid compounds were extracted following the reported method (Senevirathna et al. 2022) with modifications. Ground samples (5.0 g) were mixed with 25 mL of 80% (v/v) methanol in a 50 mL Falcon centrifuge tube, vortexed for 30 s, and centrifuged at 3000 × *g* for 10 min at 4°C (Megafuge ST4 Plus, ThermoFisher, USA). The supernatant was gently collected and stored in sealed amber tubes at −4°C in the dark until analysis.

#### 2.9.2 Total Phenolic Content

Total phenolic content (TPC) was determined using the Folin–Ciocalteu method (Jan et al. 2022). Sample extract (0.5 mL) was mixed with 0.5 mL ethanol (95%), 2.5 mL distilled water, and 2.5 mL diluted Folin–Ciocalteu reagent (1:10). After 5 min, 0.5 mL of 5% Na_2_CO_3_ was added, and the homogenous mixture was incubated in the dark for 60 min at room temperature. Absorbance was measured at 765 nm (UV–Visible spectrophotometer, Biochrom Ultrospec 7500, UK). Results were expressed as mg gallic acid equivalents per gram dry weight sample (mg GAE/g) using standard curve ranging from 50 to 300 µg/mL. The analysis was conducted in triplicate for each sample replication.

#### 2.9.3 Total Flavonoid Content

Total flavonoid content (TFC) was determined using the aluminum chloride colorimetric method (Meda et al. 2005). Extract (5 mL) was mixed with 5 mL of 2% AlCl_3_ in methanol and incubated at room temperature for 10 min. Absorbance was measured at 415 nm (UV–Visible spectrophotometer, Biochrom Ultrospec 7500, UK). Results were expressed as milligrams of quercetin equivalents per gram of dry sample (mg QE/g) using a calibration curve ranging from 5 to 40 mg/L. The analysis was conducted in triplicate for each sample replication.

#### 2.9.4 ABTS Radical‐Scavenging Activity

ABTS radical scavenging activity was determined following Wootton‐Beard et al. (2011). The ABTS radical cation (ABTS^+^) solution was prepared by reacting 7 mM ABTS with 2.45 mM potassium persulfate (1:1, v/v) and incubating in the dark for 12 h at room temperature. The solution was diluted to an absorbance of 0.70 ± 0.02 at 734 nm. Sample (0.3 mL) was mixed with 2.7 mL ABTS solution and incubated for 30 min in the dark. Absorbance was measured at 734 nm using UV–‐Visible spectrophotometer (Biochrom Ultrospec 7500, UK). The results were expressed as milligrams of Trolox equivalents per gram of dry sample (mg TE/g). The analysis was performed in triplicate for each sample replication.

#### 2.9.5 DPPH Radical Antioxidant Activity

DPPH radical scavenging activity was determined according to Brand‐Williams et al. (1995) with modifications. Sample (0.1 mL) was mixed with 3.9 mL of 80 µM DPPH solution and incubated in the dark for 30 min at room temperature. Absorbance was measured at 515 nm (UV–Visible spectrophotometer, Biochrom Ultrospec 7500, UK). Results were expressed as milligrams of Trolox equivalents per gram dry matter (mg TE/g). The measurement was performed in triplicate for each sample replication.

#### 2.9.6 Scanning of FTIR Spectra

Grounded spice extrudate breakfast cereal was analyzed by ATR‐FTIR on a Bruker Alpha II spectrometer (Bruker Optik GmbH, Germany), collecting 32 scans per spectrum at 4 cm^−1^ resolution over 4000–400 cm^−1^ with a platinum diamond ATR accessory. Nine absorbance spectra per sample served as input for correlating antioxidant activity, phenolic content, and flavonoid content with FTIR data.

#### 2.9.7 Glycemic Index

The GI of the selected SEBC was determined according to ISO 26642:2010 (International Organization for Standardization—ISO 2010). Ten healthy subjects aged 22–50 years participated after providing informed consent. The test cereal and reference glucose solution each provided 50 g of available carbohydrates. Each participant consumed the reference glucose solution on two separate occasions and the test cereal on one occasion. The mean incremental area under the curve obtained from the two reference‐food tests was used as the participant‐specific reference response. Available carbohydrate was calculated as total carbohydrate by difference minus TDF (McCleary et al. 2019).

### 2.10 Statistical Analysis

Data were obtained from two independent production replication for each formulation and are presented as mean ± standard deviation. The number of technical replications during physicochemical and functional analysis differed according to the characteristics and expected variability of each measurement; with more replicates used for physical analyses. Color, BD, and texture were measured using five replicate determinations per production run, whereas the SE index was determined from ten randomly selected extrudate pieces because of its greater dimensional variability. Cereal resistance in milk, WAI, WSI, ABTS, and DPPH radical‐scavenging activities, TFC, and TPC were measured in triplicate. TDF was determined through duplicate analytical measurements by an accredited laboratory. Technical and subsample measurements were averaged within each independent production run before inferential statistical analysis. The duplicate reference‐food tests used in the GI procedure were not treated as independent production replicates.

Differences among formulations were assessed using one‐way analysis of variance (ANOVA) (*p* < 0.05) in SPSS version 30.0. Sensory data were analyzed using XLSTAT (version 2021.2.2.1141, Addinsoft, France). RATA attributes were analyzed individually with one‐way ANOVA per attribute, followed by Duncan's multiple range test (*p* < 0.05) to compare mean citation frequencies across formulas. Hedonic liking scores followed the same ANOVA approach, but with Tukey's HSD (*p* < 0.05) used for the post‐hoc comparison of overall acceptance among formulas. FTIR‐ATR absorbance spectra analyzed with OriginPro software (Learning Edition, Origin Lab, US).

## 3 Results and Discussion

### 3.1 Proximate Content

The proximate composition of the SEBCs is presented in Table 2. Protein content did not differ significantly among formulations (*p* > 0.05). Although slightly higher values were observed in *F*2 and *F*3 than in the control, these numerical differences may reflect the contribution of protein‐containing constituents from the spice blend (protein fractions of ginger, cinnamon, and lemongrass); further research needed to confirm this hypothesis.

**TABLE 2:** Proximate analysis of spice‐enriched extruded breakfast cereal.

Formula	Proximate content				
	Moisture content (%dwb)	Ash (%dwb)	Protein (%dwb)	Fat (%dwb)	Carbohydrate (%dwb)
*F*0	6.15 ± 0.08^c^	3.32 ± 0.06^b^	5.58 ± 0.08^a^	1.71 ± 0.04^c^	83.25 ± 0.18^a^
*F*1	7.99 ± 0.03^a^	3.24 ± 0.03^b^	5.49 ± 0.08^a^	2.43 ± 0.06^b^	80.85 ± 0.09^c^
*F*2	5.98 ± 0.06^d^	3.29 ± 0.04^b^	5.79 ± 0.017^a^	2.40 ± 0.07^b^	82.55 ± 0.19^b^
*F*3	6.74 ± 0.06^b^	3.79 ± 0.08^a^	5.79 ± 0.07^a^	4.39 ± 0.11^a^	79.29 ± 0.06^d^

*Note: F*0 = formula with 0% SB—Control; *F*1 = formula with 1% SB; *F*2 = formula with 2% SB; *F*3 = formula with 3% SB.

Abbreviation: SB, Spices blend.

Moisture content was significantly affected by spice‐blend incorporation (*p* < 0.001), ranging from approximately 5.98%–7.99%, with the highest value observed in *F*1 containing 1% spice‐blend. The higher moisture content of *F*1 may be associated with differences in water retention within the starch–fiber matrix during extrusion and subsequent drying. Dietary fiber from rice bran and unripe banana flour, together with polysaccharide and phenolic constituents from the spice‐blend, may contribute water binding through hydrophilic interactions, resulting in a denser, less expanded structure (Tyl et al. 2021; Renoldi et al. 2021). However, the absence of a concentration‐dependent increase indicates that moisture retention was probably influenced by the combined effects of formulation, expansion, and drying behavior rather than spice concentration alone.

Fat content increased significantly with increasing spice concentration (*p* < 0.001), reaching the highest value in *F*3 at 4.39. This trend may reflect the contribution of lipid‐soluble constituents, including oleoresins and essential‐oil components in spices, which can be counted within the crude‐fat fraction during proximate analysis (Singh et al. 2007; Puspitojati et al. 2025). Ash content also increased significantly at the higher spice levels (*p* = 0.001), which is consistent with the additional mineral contribution of the spice ingredients (Arshad et al. 2025).

In contrast, carbohydrate content decreased significantly as spice‐blend concentration increased (*p* < 0.001), with the control showing the highest value of 83.25%. Because carbohydrate was calculated by difference, this decrease was partly attributable to the proportional increases in moisture, fat, ash, and other non‐carbohydrate constituents rather than necessarily to direct carbohydrate degradation (Okafor and Falade 2025; Sarmiento‐Torres et al. 2025).

The TDF content of the optimized formulation containing 1% spice‐blend was 5.65%, approximately 30% lower than the theoretical value of 8.03% estimated from the raw‐material composition (Table 3). Because the theoretical value was calculated from ingredient data rather than measured in the unprocessed mixture, this may be interpreted as an indication of possible processing‐related changes rather than as a direct fiber mass balance. The lower measured TDF may be partly associated with structural modifications occurring during extrusion. High temperature, pressure, and shear can depolymerize insoluble polysaccharides, including cellulose and hemicellulose, into lower‐molecular‐weight fractions that may not be completely recovered by enzymatic–gravimetric methods used in the study (Yang et al. 2017; Ain et al. 2019). Resistant starch type 2 from unripe banana flour may also undergo gelatinization during extrusion, increasing its enzymatic digestibility and reducing its contribution to measured dietary fiber (Baptista et al. 2024; Z. Wang et al. 2023).

**TABLE 3:** Estimated Raw‐Material Contributions to Total Dietary Fiber in Spice‐Enriched Extruded Cereal.

Raw material	%	Dietary fiber (%)	Source
Corn starch	55	3.85	Packaging
Rice bran	20	0.97	Widowati et al. (2020)
Banana flour	10	3.11	Alam et al. (2015)
Salt	1	0	—
Water	5	0	—
Skim milk	5	0	—
Palm sugar	3	0	—
Ginger	0.75	0,11	U.S. Department of Agriculture, Agricultural Research Service (2019)
Cinnamon	0.2	0.06	Senevirathne et al. 2022
Lemongrass	0.05	0.03	Villalobos et al. 2021
Estimated TDF 1% SB		8.03%	
The result analysis TDF 1%SB		5.65%	

Interactions between spice‐derived polyphenols and dietary fiber components may also affect analytical recovery for TDF; however, this mechanism was not directly verified in the present study. Polyphenols can associate with dietary fiber (polysaccharides in general) through hydrogen bonding and hydrophobic interactions, potentially influencing their behavior during enzymatic digestion and ethanol precipitation (Jakobek 2015; Saura‐Calixto 2011). The observed total phenolic and flavonoid contents confirm the presence of extractable bioactive compounds but do not, by themselves, demonstrate polyphenol–fiber complex formation.

In this study, the contribution of retrograded starch may also have been limited. Previous studies have reported increased apparent TDF in extruded rice bran–banana flour products through the formation of resistant starch type 3 during cooling (Haralampu 2000). In the present study, hot‐air drying immediately after extrusion may have restricted the time available for starch retrogradation, thereby limiting the contribution of newly formed resistant starch to the measured TDF (Haralampu 2000). Nevertheless, resistant‐starch fractions and starch crystallinity were not directly measured in this study; further research needed to confirm this plausible hypothesis. Although the measured TDF was lower than the theoretical estimate, extrusion may alter the distribution of insoluble and soluble fiber fractions, which is further can be associated with improved physiological effects, including glycemic regulation and cholesterol reduction (Mironeasa et al. 2023). The increase in WSI from 6.34% to 8.55% (Table 3) at higher spice levels is consistent with greater solubilization or fragmentation of matrix components. However, WSI is not a direct measure of soluble dietary fiber and should not be used alone to infer improved physiological functionality. Direct determination of soluble and insoluble dietary fiber, resistant‐starch fractions, molecular‐weight distribution, and structural characteristics would be required further research to confirm the nature and nutritional significance of these transformations.

### 3.2 Physical Properties

#### 3.2.1 Color

**FIGURE 1:**
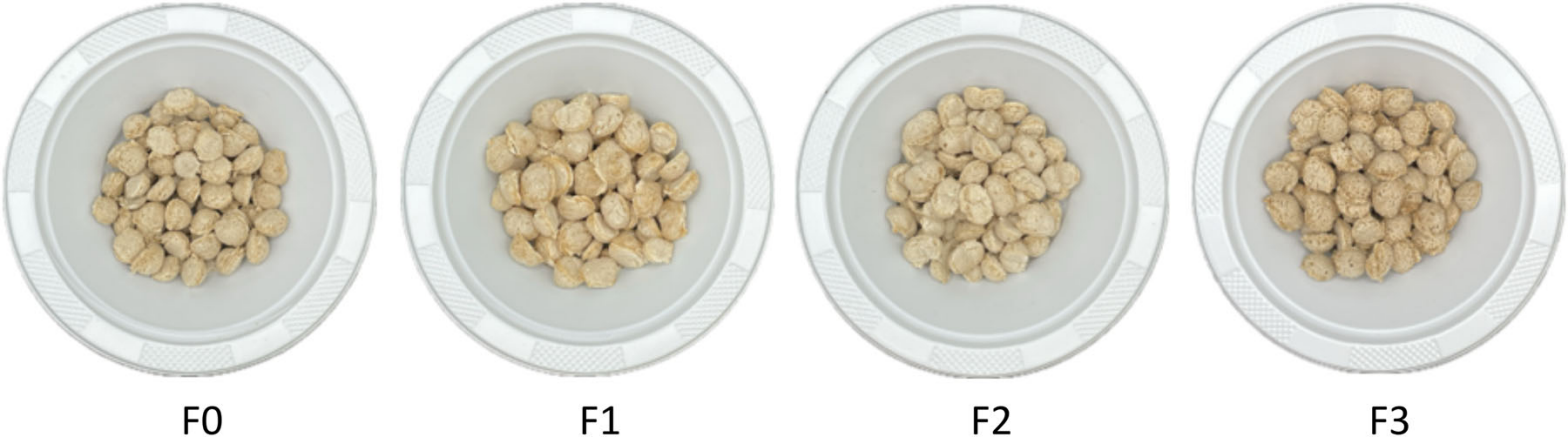
Visual of spice‐enriched extruded breakfast cereal for Control = formula with 0% SB; *F*1 = formula with 1% SB; *F*2 = formula with 2% SB; *F*3 = formula with 3% SB; SB = Spices blend.

Spice‐blend incorporation significantly affected all color parameters (*p* < 0.001) (Table 4). Lightness (L*) increased from 65.07 in the control *F*0 to 70.50–71.47 in spice‐blend formulations *F*1–*F*3 (*compare to* Figure 1). Because the proportions of corn starch, rice bran, and banana flour were maintained across formulations, this increase might not be attributed to dilution by the base ingredients. Instead, it may more reflect the combined effects of spice‐derived pigments, differences in product expansion and surface structure, and increased light scattering; or the interaction between spice‐derived pigments and the base ingredients materials. This increase was likely due to dilution of corn carotenoids by the lighter rice bran–banana flour matrix and enhanced expansion, which increased light scattering (Kaur et al. 2015).

**TABLE 4:** Physical properties of spiced‐enriched extruded breakfast cereal.

Parameter	Formula			
	*F*0	*F*1	*F*2	*F*3
Resistance of cereal in milk (min)	26.35 ± 0.19^b^	27.26 ± 0.21^a^	27.63 ± 0.4^a^	24.67 ± 0.61^c^
Hardness (N)	22.68 ± 0.87^a^	22.27 ± 0.90^a^	13.53 ± 0.40^c^	16.02 ± 1.35^b^
Crunchiness (Nmm)	49.52 ± 1.48^b^	50.31 ± 1.02^ab^	22.99 ± 0.76^c^	52.34 ± 2.34^a^
BD (g/mL)	0.0916 ± 0.002^b^	0.1016 ± 0.004^b^	0.0942 ± 0.003^a^	0.1031 ± 0.002^a^
WAI (g/g)	4.30 ± 0.31^b^	4.90 ± 0.18^a^	4.80 ± 0.07^a^	5.03 ± 0.21^a^
WSI (%)	7.12 ± 0.68^b^	6.34 ± 0.03^c^	6.67 ± 0.20^bc^	8.55 ± 0.23^a^
SE (mm)	2.68 ± 0.02^b^	2.56 ± 0.07^c^	2.52 ± 0.09^c^	2.79 ± 0.12^a^
Color				
L*	65.07 ± 1.97^b^	70.50 ± 0.98^a^	71.22 ± 0.13^a^	71.47 ± 0.44^a^
a*	2.51 ± 0.09^b^	1.69 ± 0.05^c^	2.633 ± 0.10^b^	3.93 ± 0.07^a^
b*	15.93 ± 0.66^b^	13.3 ± 0.42^c^	16.062 ± 0.38^b^	18.59 ± 0.30^a^
c*	16.13 ± 0.64^b^	13.41 ± 0.41^c^	16.30 ± 0.39^b^	19.01 ± 0.28^a^
h*	81.04 ± 0.67^b^	82.76 ± 0.43^a^	80.30 ± 0.14^b^	78.06 ± 0.33^c^
∆*E*	67.04 ± 2.06^b^	71.76 ± 0.88^a^	73.15 ± 0.06^a^	73.95 ± 0.49^a^

*Note: F*0 = formula with 0% SB—Control; *F*1 = formula with 1% SB; *F*2 = formula with 2% SB; *F*3 = formula with 3% SB.

Abbreviation: SB, Spices blend.

Redness (a*) and yellowness (b*) initially decreased at 1% SB and subsequently increased at 2%–3% spice‐blend, indicating a non‐linear color response (Table 4). This response may result from the combined contribution of pigments naturally present in the spice blend, that intensified non‐enzymatic browning, and thermally induced reactions during extrusion (Maillard reaction). Maillard reactions between reducing sugars and amino‐containing compounds may contribute to the formation of brown‐colored products, while thermal degradation or transformation of ginger, cinnamon, and lemongrass pigments may further affect the final color (Schutte et al. 2024; Krahl et al. 2016; Szymczak et al. 2026; Assiéné Agamou et al. 2024; Hashim et al. 2019). Nevertheless, individual pigments and Maillard‐reaction products were not quantified; further research need to be done to confirm their contributions.

Chroma (C*) increased with spice‐blend concentration and reached its highest value at 3% spice‐blend, indicating greater color intensity (Table 4, *compare to* Figure 1). Meanwhile, the hue angle decreased from 82.76 to 78.06, reflecting a shift toward more reddish‐yellow tones. The total color difference (Δ*E*) also increased progressively, indicating that the spice‐enriched formulations were increasingly distinguishable from the control and developed a more pronounce golden‐brown appearance. This natural color development may contribute to product differentiation as a SEBC (Žilić et al. 2022; Sebastião et al. 2023); however, consumer perceptions of naturalness or “clean‐label” characteristics were not directly evaluated in this study.

#### 3.2.2 Cereal Resistance in Milk

Cereal resistance in milk differed significantly among formulations (*p* < 0.001) (Table 4). The formulations containing 1% and 2% spice‐blend exhibited the longest resistance times, at 27.26 and 27.63 min, respectively, compared with 26.35 min for the control *F*0 and 24.67 min for the *F*3 with 3% spice‐blend formulation. The improved resistance at moderate spice‐blend concentration may be associated with differences in matrix compactness, pore structure, and the integrity of the cell walls, developed by starch‐protein interaction; which could delay milk penetration and structural softening. Although hydrogen bonding and hydrophobic starch–protein interactions have been reported to strengthen extruded matrices (Smietana et al. 1988), the present data do not directly confirm this mechanism in the spice‐enriched extruded cereal. Further studies are needed to elucidate the underlying interactions.

The reduction in resistance at 3% spice‐blend may indicate that the higher spice concentration alters or disrupting matrix continuity, increasing porosity and facilitating liquid penetration. Fiber, soluble constituents, and lipid‐like compounds from the spice blend may have contributed to this response (Masavang et al. 2019; Menis‐Henrique et al. 2020); however, direct microstructural evidence was not obtained. All formulations exceeded the previously reported minimum resistance time of 3 min for ready‐to‐eat breakfast cereals (Gregson and Lee 2002). Thus, moderate spice blend incorporation improved crispness retention in milk, whereas the higher spice blend levels reduced resistance relative to the other spice‐enriched formulation.

#### 3.2.3 Texture

Spice‐blend incorporation significantly affected hardness and crispness/crunchiness in this study (*p* < 0.001) (Table 4). These properties are important determinants of extruded‐cereal acceptability and are generally related to product expansion and internal “cell structure.” Desirable extruded breakfast cereals are commonly characterized by relatively low hardness and a distinct crisp or crunchy fracture response (Sharma et al. 2019).

Hardness was similar between the control *F*0 and *F*1 with 1% spice blend formulation, with values of 22.68 and 22.27 N, respectively (Table 4). It decreased markedly to 13.53 N at 2% spice‐blend concentration for *F*2 and subsequently increased to 16.02 N at 3% spice‐blend for *F*3. This non‐linear response may reflect changes in expansion, pore distribution, and cell‐wall strength caused by spice‐blend incorporation. The lower hardness at 2% SB is consistent with the possibility of a more easily fractured structure, although matrix porosity was not directly measured (Renoldi et al. 2021; Charunuch et al. 2011).

The partial increase in hardness at 3% spice‐blend (Table 4) may indicate the formation of a comparatively more compact or mechanically resistant structure, consistent with its higher BD due to increase of fiber content. However, the relationship between BD, SE, and hardness was not strictly linear, indicating that these parameters were influenced by several interacting formulation and processing factors (Guevara Guerrero et al. 2019).

Crunchiness or crispness reached its highest value at *F*3 with 3% spice‐blend formulation at 52.34 N·mm (Table 4). This higher value may reflect a greater fracture response during compression, but it should not be interpreted solely as evidence of a denser matrix without supporting microstructural analysis; further study is needed to prove this (Philipp et al. 2017). Spice‐derived phenolic compounds may interact with starch or protein components and influence gelatinization behavior and matrix formation due to alteration of cell wall rigidity during extrusion; however, these interactions were not directly demonstrated in the present study (Borah et al. 2016). The findings demonstrate a non‐linear textural response, with 2% spice‐blend producing the lowest hardness and 3% spice‐blend producing the highest measured crispness or crunchiness.

#### 3.2.4 WAI and WSI

Spice‐blend incorporation significantly affected the WAI (*p* < 0.05) and WSI (*p* < 0.001) (Table 4). WAI increased from 4.30 g/g in the *F*0 to 4.80–5.03 g/g in the spice‐enriched formulations (*F*1–*F*3). The higher WAI may reflect an increased capacity of the extruded matrix to retain water because of the combined contributions of gelatinized starch and hydrophilic fiber or polysaccharide constituents from the spice blend (Borah et al. 2016; Yadav et al. 2024). Polyphenol‐starch interaction may also affect water absorption and swelling behavior (Jaworska et al. 2019); however, the present results do not provide direct evidence of such molecular interactions.

WSI showed a non‐linear trend, decreasing to 6.34%–6.67% at *F*1–*F*2 and increasing to 8.55% at *F*3 (Table 4). The lower WSI at moderate spice‐blend levels indicates a smaller proportion of water‐soluble material than in the control *F*1. This may be associated with reduced starch fragmentation, dilution of starch or fiber by other constituents, or incorporation of less‐soluble material into the matrix. In contrast, the higher WSI at 3% SB may reflect greater solubilization of matrix components, increased macromolecular fragmentation, or the contribution of water‐soluble constituents from the spice blend (Alam et al. 2015; Gutkoski and El‐Dash 1999).

Because WSI does not distinguish between soluble starch fragments and soluble compounds originating from the spice blend, the increase at 3% spice blend (*F*3) cannot be attributed exclusively to extrusion‐induced matrix degradation. However, these results suggest moderate SB levels increased water absorption while maintaining structural integrity, whereas the 3% SB formulation showed the greatest release of soluble material.

#### 3.2.5 SE and BD

Spice‐blend concentration significantly influenced both SE and BD (*p* < 0.001) (Table 4). These parameters, SE and BD, describe complementary aspects of the internal structure and expansion behavior of extruded products.

BD increased from 0.0917 g/mL in the control *F*0 to 0.1031 g/mL at 3% spice‐blend *F*3 (Table 4), indicating the formation of a more compact product at the highest spice concentration. The increase in BD may be associated with the incorporation of fiber and other non‐starch components that reduced the continuity of the starch phase and limited overall expansion. Although changes in melt viscosity have been proposed to explain similar responses, melt rheology was not measured in the present study (Bazán‐Colque et al. 2023). Similar increases in density have been reported following the incorporation of bran and other plant‐derived ingredients into extruded cereal products (Delgado‐Nieblas et al. 2019).

SE decreased from 2.68 in the control *F*0 to its lowest value in of 2.52 mm in *F*2 (Table 4), suggesting that moderate spice‐blend incorporation restricted radial expansion, due to disruption of the starch matrix by fiber, limiting vapor‐driven expansion (Borah et al. 2016). Fiber‐rich ingredients may interrupt the continuity of the starch matrix and limited vapor‐driven expansion at the die exit (Borah et al. 2016; Mridula et al. 2017). However, SE increased at 3% spice‐blend, suggesting that the relationship between spice concentration and expansion was non‐linear.

The higher SE at 3% spice‐blend concentration may reflect the combined effects of moisture distribution, lipid‐soluble spice constituents, fiber concentration, and changes in melt behavior. Essential‐oil components could theoretically exert a plasticizing effect within the extruded matrix, but this mechanism cannot be confirmed without direct rheological, thermal, or structural characterization. Concentration‐dependent effect of spice‐blend incorporation on expansion and density is need to be confirmed using the advanced structural analysis.

### 3.3 Sensory Properties

The demographic characteristics of the participants or panelists involved in this study are presented in Table 5. The 70 untrained panelists, 77.1% were aged 19–25, 72.9% were female, and 91.4% were students, with most residing in the Greater Jakarta area and West Java. Older adults, male consumers, and those from outside Java were underrepresented. Thus, the sensory and hedonic findings should be interpreted as reflecting the responses of the recruited consumer group (refer to SNI 01‐2346‐2006). Validation using a larger and more demographically diverse consumer sample is recommended for further study.

**TABLE 5:** Demographic panelist for sensory evaluation of the spice‐enriched extruded breakfast cereal (*n* = 70).

Variable	Class	Frequency (*n*)	Percentage (%)
Gender	Female	51	72.9
	Male	19	27.1
Age group (years)	19–25	54	77.1
	26–35	15	21.4
	36–45	1	1.4
Professional status	Student	64	91.4
	Lecturer	2	2.9
	Researcher	2	2.9
	Laboratory Assistant	2	2.9
Geographic origin	West Java	23	32.9
	Central Java	12	17.1
	East Java	7	10.0
	Jakarta	5	7.1
	South Sumatra	5	7.1
	West Sumatra	3	4.3
	Riau	3	4.3
	Banten	2	2.9
	South East Sulawesi	2	2.9
	South Sulawesi	1	1.4
	Lampung	1	1.4
	North Sumatra	1	1.4
	Aceh	1	1.4
	South Kalimantan	1	1.4
	West Nusa Tenggara	1	1.4
	Japan	1	1.4
	Kenya	1	1.4

#### 3.3.1 Check‐All‐That‐Apply

The CATA evaluation conducted under three serving conditions (dry cereal, cereal with cold fresh milk, and cereal with warm fresh milk) showed that spice‐blend concentration tested significantly influenced multiple sensory attributes (Table 6) (*p* < 0.001). Of the 25 attributes evaluated using Cochran's *Q* test, significant differences among formulations were observed primarily for aroma, taste, mouthfeel, and aftertaste attributes, whereas most texture‐related attributes did not differ significantly (Table 6). These results indicate that spice‐blend incorporation primarily differentiated the formulations through flavor‐related perceptions. The absence of significant differences in most texture attributes suggests that consumers perceived the samples as having broadly similar textural characteristics under the evaluated conditions; however, it does not confirm that spice‐blend incorporation caused no structural changes in the extruded matrix (Proserpio et al. 2020; Dias‐Faceto and Conti‐Silva 2022).

**TABLE 6:** Counts of check‐all‐that apply (CATA) of spice‐enriched extruded breakfast cereal for each serving conditions.

Attributes sensory	Ideal	Non serving milk					Cold milk serving					Warm milk serving				
		*F*0	*F*1	*F*2	*F*3		*F*0	*F*1	*F*2	*F*3		*F*0	*F*1	*F*2	*F*3	
Cream C.	64	67	69	69	67	^ns^	67	69	69	69	^ns^	70	67	68	69	^ns^
Sweet A.	54	52	49	58	61	^**^	54	60	58	61	^ns^	54	56	57	56	^ns^
Cinnamon A.	24	47	64	55	61	^***^	60	49	61	59	^**^	61	56	51	59	^*^
Spicy A.	1	24	53	45	43	^***^	48	27	49	44	^***^	48	41	36	51	^**^
Toasted A.	43	66	60	62	65	^ns^	60	64	59	66	^ns^	61	62	66	64	^ns^
Branny A.	49	66	60	62	65	^ns^	60	68	61	63	^*^	63	65	67	62	^ns^
Ginger A.	11	37	61	53	54	^***^	53	36	56	55	^***^	55	56	44	61	^***^
Starchy A.	31	65	57	59	61	^ns^	59	65	59	57	^ns^	57	60	62	54	^ns^
Musty A.	4	37	42	48	47	^*^	39	40	44	41	^ns^	39	45	50	44	^*^
Earthy A.	8	39	42	40	47	^ns^	41	43	38	39	^ns^	35	46	51	41	^***^
Milky A.	57	49	30	67	66	^***^	34	63	65	66	^***^	33	64	59	62	^***^
Salty T.	33	60	63	63	66	^ns^	63	65	59	61	^ns^	63	64	63	62	^ns^
Sweet T.	66	51	45	50	54	^ns^	50	59	50	52	^ns^	47	57	55	50	^*^
Bitter T.	9	49	67	59	55	^***^	58	41	63	57	^***^	64	56	57	64	^*^
Umami T.	23	53	51	57	60	^*^	53	54	53	56	^ns^	52	54	56	54	^ns^
Spicy F.	33	32	63	63	54	^***^	63	37	61	58	^***^	65	57	45	61	^***^
Crunchy Tx.	63	70	70	70	70	^ns^	70	70	70	70	^ns^	70	70	68	70	^ns^
Soluble Tx.	28	64	67	68	70	^*^	65	66	69	70	^*^	66	68	68	69	^ns^
Tooth Packed Tx.	12	63	65	66	66	^ns^	64	66	67	67	^ns^	67	66	68	67	^ns^
Brittle Tx.	47	69	69	69	67	^ns^	69	69	70	67	^ns^	70	69	69	69	^ns^
Salty Af.	24	61	56	57	63	^ns^	60	59	55	57	^ns^	60	58	60	59	^ns^
Sweet Af.	65	49	34	42	49	^ns^	41	53	38	46	^ns^	39	49	50	41	^ns^
Spicy Af.	2	29	60	56	53	^***^	58	29	58	58	^***^	60	52	35	59	^**^
Warmth Af.	27	33	59	56	58	^***^	58	33	55	59	^***^	62	49	51	61	^***^
Bitter Af.	6	45	66	63	59	^***^	61	43	65	63	^***^	64	55	53	68	^***^

*Note*: ns *p* ≥ 0.05. C = Color; A = Aroma; T = Taste; Tx = Texture; Af = Aftertaste. *F*0 = formula with 0% SB—Control; *F*1 = formula with 1% SB; *F*2 = formula with 2% SB; *F*3 = formula with 3% SB.

Abbreviation: SB, Spices blend.

***p < 0.001; **p < 0.01; *p < 0.05.

Aroma was among the most discriminating sensory modalities. Ginger and spicy aromas differed significantly among formulations under all three serving conditions (*p* < 0.001). This finding is consistent with the contribution of volatile and semi‐volatile compounds derived from ginger and the other spices, although individual aroma compounds were not measured in the present study (Semwal et al. 2015). Cinnamon aroma differentiated the formulations less consistently after milk was added, indicating that the milk matrix may have modified the release or perception of cinnamon‐derived volatiles (might be as partial masking) (Pramudya and Seo 2018); however, this volatile partitioning was not directly evaluated. Further studies are needed to confirm the mechanism.

Milky aroma differed significantly under all serving conditions, whereas sweet aroma was significant only for the dry‐cereal condition (*p* < 0.001). The addition of milk may have reduced the relative perceptual contrast in sweet aroma among the formulations by introducing a common dairy aroma and taste background. Earthy aroma became significant under the warm‐milk condition (*p* < 0.001). The warmer serving temperature may have increased the release of some bran‐ or spice‐associated volatile compounds (Heiniö et al. 2003); however, the specific compounds responsible for this perception were not identified.

Taste and mouthfeel attributes showed comparable formulation‐dependent responses. Spicy sensation differed significantly (*p* < 0.001) among formulations under all serving conditions, consistent with the increasing contribution of pungent compounds from the spice blend. Ginger‐derived compounds, including gingerols (i.e., 6‐gingerol) and shogaols, are known to elicit trigeminal sensations, although receptor (TRPV1 receptor) activation was not directly assessed in this study (Semwal et al. 2015).

Bitter taste differentiated the formulations less strongly after the addition of milk. This reduction of perception may be related to dilution, flavor masking, or partitioning of some hydrophobic taste‐active compounds into the milk matrix, but the relative contribution of these processes was not determined (Des Gachons et al. 2012). Umami taste differed significantly (*p* < 0.05) only under the dry‐cereal condition, suggesting that this relatively weak distinction became less apparent in the presence of milk (might be due to masking by the dairy flavor).

Most texture attributes, including crunchy, tooth‐packing, and brittle, did not differ significantly among formulations in the CATA analysis. This indicates that texture was not a major source of consumer‐perceived differentiation among the samples, confirming that spice incorporation up to 3% did not disrupt the extruded starch network governed by process parameters rather than minor additions (Brennan et al. 2013). Spicy, bitter, and warming aftertastes differed significantly (*p* < 0.001 to *p* < 0.01) among formulations across the serving conditions, indicating persistent flavor‐active compounds of nature of gingerols, cinnamaldehyde, and citral which bind to oral receptors and create prolonged sensory impressions.

**FIGURE 2:**
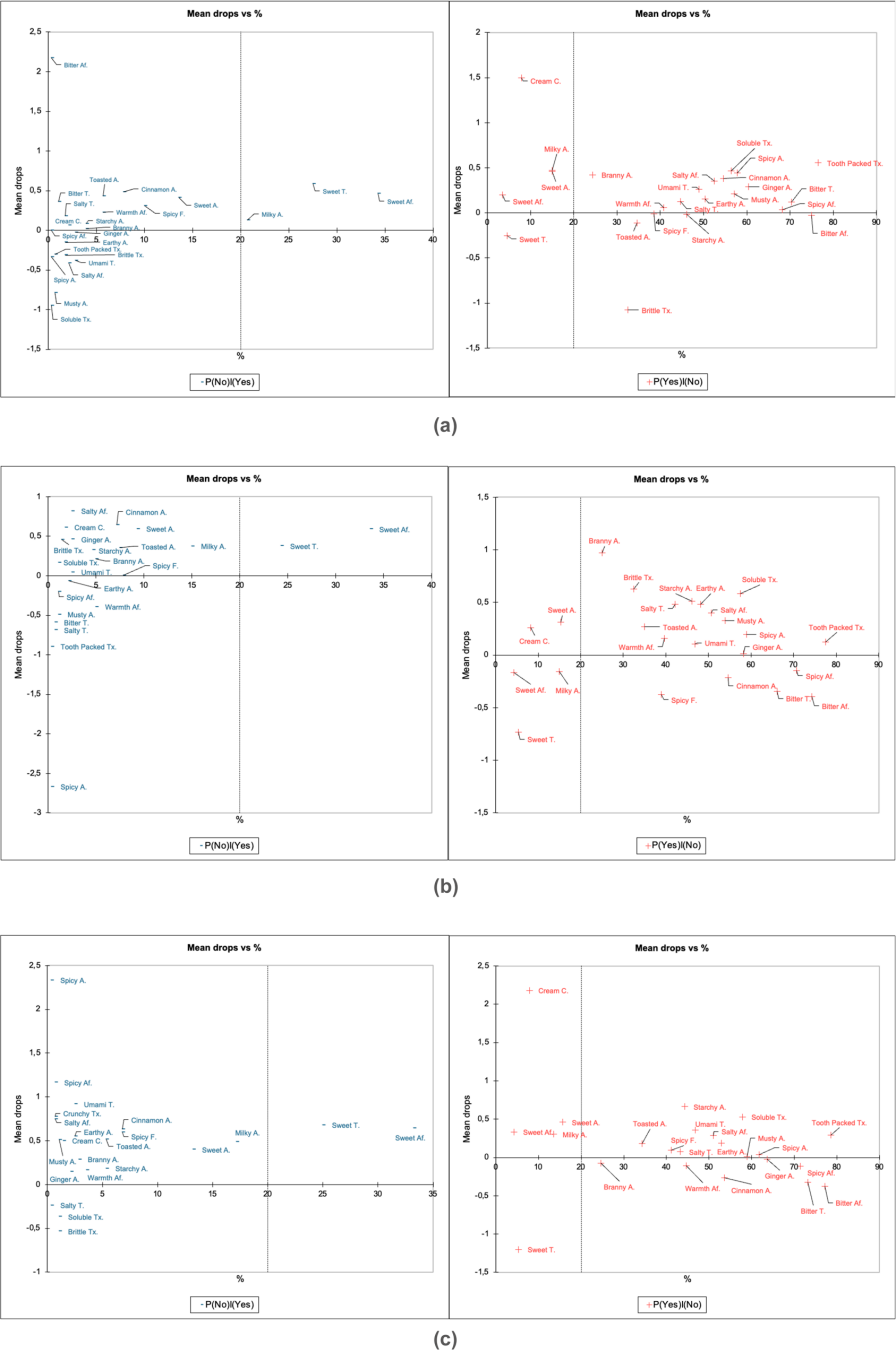
Penalty analysis of spice extruded cereal for each serving conditions. (a) non milk serving (b) cold milk serving (c) warm milk serving.

Penalty analysis based on the CATA data (Figure 2) identified sweet taste as the most positive driver of liking. Its absence produced mean drops in overall liking of 0.59 for dry cereal, 0.38 for cereal with cold fresh milk, and 0.68 for cereal with warm fresh milk (*p* < 0.05). This finding indicates that sweetness was an expected characteristic of the breakfast cereal and that samples not perceived as sweet were rated less favorably.

Cream color and milky aroma identified as “must‐have” attributes, showed positive effects on liking when selected (high % selection, positive mean drop, particularly in the P(Yes)I(No) plots), suggesting that these attributes were consistent with consumers’ expectations of a breakfast cereal, particularly when served with milk. In contrast, the presence of spicy aroma and bitter aftertaste was associated with reductions in overall liking, occupied of the P(No)I(Yes) plots with a high negative mean drop (−2.7 to −3.0). Because CATA records attribute occurrence rather than intensity, these findings indicate that consumers who perceived these attributes tended to give lower liking scores; they do not, by themselves, demonstrate an intensity‐dependent effect (Proserpio et al. 2020).

Bitter aftertaste was consistently located in the penalty region across serving conditions, indicating that it was an important negative driver of acceptance. This perception may be associated with bitter compounds originating from rice bran, unripe banana flour, and the spice blend; however, the compounds responsible were not chemically identified. Texture attributes, including crunchy, brittle, tooth‐packing, and soluble, were positioned close to a zero‐mean drop. Thus, although these attributes contributed to the sensory profile of the cereals, their presence or absence had a comparatively limited effect on overall liking within the evaluated consumer group.

#### 3.3.2 Rate‐All‐That‐Apply

One‐way ANOVA was conducted for each RATA attribute prior to PCA, examining the effects of product formulation (spice blend concentration: 0%–3%) and consumer (individual panelist) on intensity ratings within each serving condition (Table 7). Consumer was included in the model to account for individual differences in scale use and sensory sensitivity among the untrained participants. The consumer effect was significant (*p* < 0.001) for all 25 attributes under three serving conditions, confirming substantial inter‐individual variation in perceived attribute intensity.

**TABLE 7:** ANOVA independent and observed variables rate‐all‐that‐apply (RATA) of spice‐enriched extruded breakfast cereal for each serving conditions.

Attributes sensory	Non serving milk		Cold milk serving		Warm milk serving	
	Product	Consumer	Product	Consumer	Product	Consumer
Cream C.	1.61^ns^	3.95^***^	1.67^ns^	5.27^***^	6.46^***^	2.56^***^
Sweet A.	3.09^*^	4.08^***^	2.71^*^	4.35^***^	5.93^***^	5.52^***^
Cinnamon A.	22.58^***^	2.34^***^	21.37^***^	3.46^***^	6.68^***^	3.92^***^
Spicy A.	24.91^***^	4.79^***^	13.71^***^	5.61^***^	10.21^***^	5.74^***^
Toasted A.	0.66^ns^	6.20^***^	1.43^ns^	7.21^***^	11.18^***^	6.57^***^
Branny A.	4.08^**^	4.35^***^	11.27^***^	5.01^***^	18.85^***^	7.66^***^
Ginger A.	34.04^***^	4.35^***^	19.85^***^	4.90^***^	9.42^***^	4.11^***^
Starchy A.	5.54^**^	4.97^***^	8.36^***^	5.48^***^	4.68^**^	8.00^***^
Musty A.	5.23^**^	5.16^***^	3.45^*^	6.78^***^	5.20^**^	7.21^***^
Earthy A.	2.71^*^	6.32^***^	0.71^ns^	9.23^***^	12.00^***^	9.22^***^
Milky A.	50.38^***^	2.56^***^	49.85^***^	2.99^***^	41.20^***^	3.27^***^
Salty T.	0.83^ns^	4.56^***^	4.51^**^	6.43^***^	2.27^ns^	5.48^***^
Sweet T.	4.72^**^	6.45^***^	3.32^*^	5.62^***^	3.96^**^	6.22^***^
Bitter T.	25.46^***^	2.97^***^	19.26^***^	3.45^***^	10.41^***^	3.18^***^
Umami T.	3.22^*^	9.33^***^	1.75^ns^	12.20^***^	1.82^ns^	10.56^***^
Spicy F.	51.70^***^	3.73^***^	39.59^***^	3.90^***^	29.36^***^	3.47^***^
Crunchy Tx.	13.01^***^	2.61^***^	3.65^*^	3.44^***^	18.43^***^	3.01^***^
Soluble Tx.	2.89^*^	3.00^***^	12.33^***^	3.70^***^	6.34^***^	3.96^***^
Tooth Packed Tx.	1.03^ns^	3.60^***^	1.86^ns^	5.82^***^	0.73^ns^	3.96^***^
Brittle Tx.	7.90^***^	3.81^***^	5.12^**^	5.34^***^	2.55^ns^	5.79^***^
Salty Af.	2.89^*^	5.32^***^	2.45^ns^	5.17^***^	3.90^**^	6.29^***^
Sweet Af.	6.89^***^	6.44^***^	2.73^*^	5.89^***^	3.32^*^	7.70^***^
Spicy Af.	58.64^***^	4.58^***^	50.24^***^	4.80^***^	44.00^***^	5.06^***^
Warm Af.	36.53^***^	4.86^***^	39.78^***^	5.48^***^	18.42^***^	4.57^***^
Bitter Af.	30.66^***^	2.91^***^	22.53^***^	3.69^***^	12.28^***^	3.62^***^

*Note*: ns *p* ≥ 0.05. C = Color; A = Aroma; T = Taste; Tx = Texture; Af = Aftertaste.

***p < 0.001; **p < 0.01; *p < 0.05.

The product effect was more selective. Of the 25 attributes, 21 differed significantly (*p* < 0.05) when the cereal was evaluated dry and with warm milk, whereas 19 attributes differed significantly under the cold‐milk condition (Table 7). Tooth‐packing texture was the only attribute that did not differ significantly among formulations under any serving condition. The strongest product discrimination (*p* < 0.001 across all serving conditions) was observed for spicy sensation, spicy aftertaste, warming aftertaste, bitter aftertaste, milky aroma, ginger aroma, cinnamon aroma, and bitter taste. These significant attributes were given particular consideration in the subsequent PCA interpretation.

**FIGURE 3:**
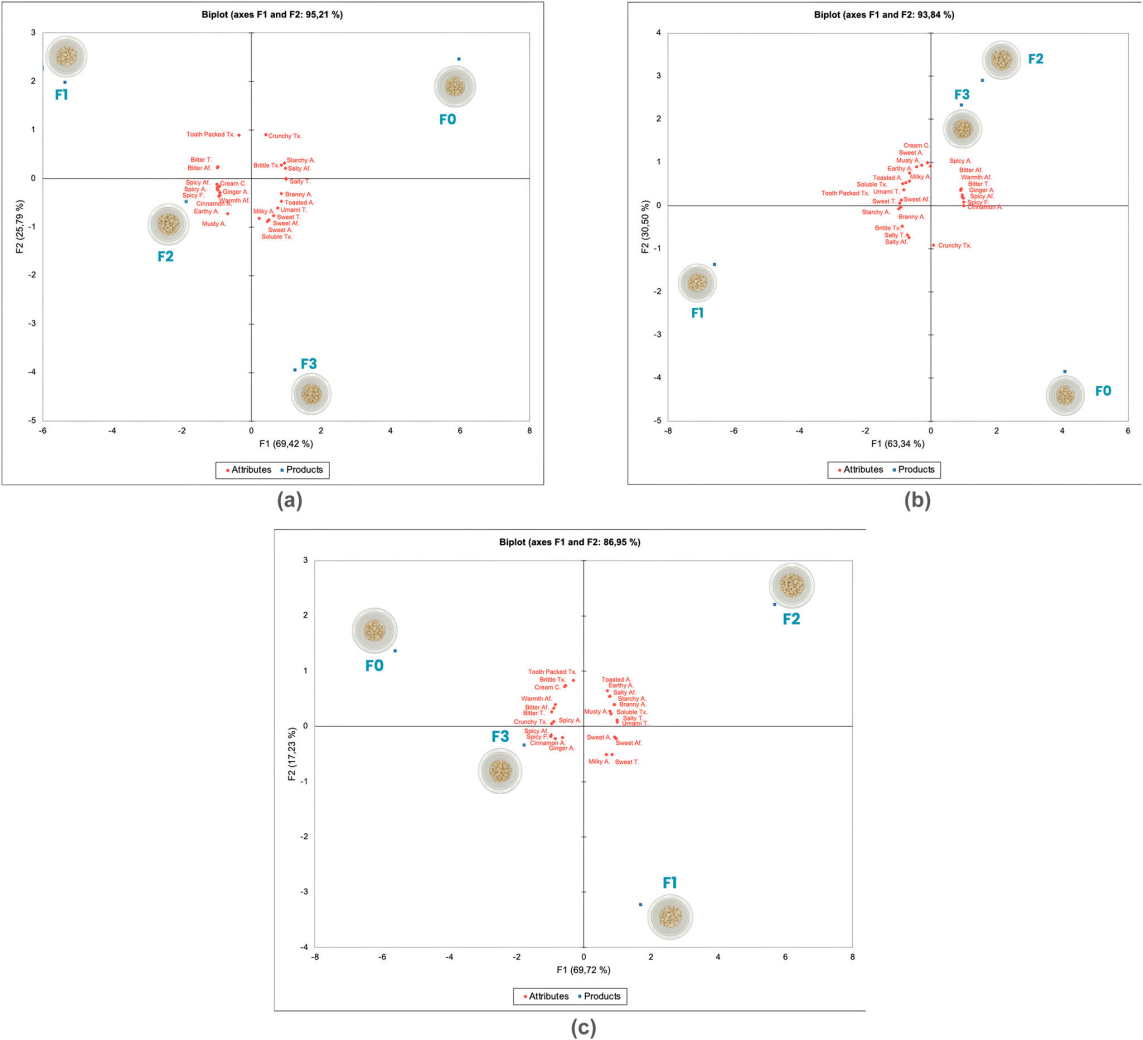
PCA Biplot RATA based consumer panel (*n* = 70) on different condition serving (a) non milk serving (b) cold milk serving (c) warm milk serving for Control = formula with 0% SB; *F*1 = formula with 1% SB; *F*2 = formula with 2% SB; *F*3 = formula with 3% SB; SB = Spices blend.

The PCA of the mean RATA intensity ratings is presented in Figure 3. The first two principal components (*F*1 and *F*2) explained 86.95%–95.21% of the total variance, indicating that the two‐dimensional configurations adequately summarized most of the sensory variation among formulations under each serving condition. Under the dry cereal conditions, the formulations containing 1% (*F*1) and 2% (*F*2) were associated with attributes including bitter taste, spicy aroma, ginger aroma, earthy aroma, bitter aftertaste, and warming aftertaste. In contrast, the control *F*0 formulation was more closely associated with starchy aroma and crunchy texture. The 3% spice‐blend formulation was clearly separated from the other samples, indicating that the highest spice concentration generated a distinct combination of sensory intensities rather than a simple linear intensification of the profile observed at lower concentrations. Thus, each spice level produced a distinguishable sensory configuration when the cereal was consumed without milk (Næs et al. 2021).

Under the cold‐milk condition, the relative positions of the samples changed, and the 2% (*F*2) and 3% (*F*3) spice‐blend formulations were located more closely together confirming reduced sensory differentiation. This finding indicating that the addition of cold milk reduced the perceived differences between the higher‐spice formulations. The milk matrix may have altered the release, dilution, or perception of aroma and taste compound (Machado et al. 1999; Sacchetti et al. 2005), although volatile partitioning and compound‐specific interactions with milk fat were not directly measure in the present study. The *F*1 with 1% spice blend incorporation remained separated from the other samples, indicating that it retained a distinct and comparatively balanced sensory profile in cold milk.

Under the warm‐milk condition, the formulations again showed a different spatial distribution. The control *F*0 and *F*3 with 3% spice‐blend incorporation were positioned on the negative side of PC1 (left side) and were associated with tooth‐packing texture, brittle texture, cream color, warming aftertaste, and bitter aftertaste. The *F*2 with 2% spice‐blend occupied the upper‐right quadrant and was associated with toasted aroma, earthy aroma, salty aftertaste, branny aroma, and soluble texture. In contrast, the *F*1 with 1% spice‐blend incorporation was located in the lower‐right quadrant near sweet aroma, milky aroma, and sweet taste.

The cumulative variance explained by the first two components decreased from 95.21% under the dry condition to 86.95% under the warm‐milk condition, suggesting a more multidimensional sensory space influenced by temperature. This lower proportion indicates that the sensory variation under warm‐milk serving was distributed across a greater number of dimensions and therefore was not captured as completely by the first two principal components. The warmer serving medium may have modified aroma release, hydration, matrix softening, and trigeminal perception, thereby altering the relative intensities and relationships among attributes. These findings were consistent with previous study that elevated temperature enhances Maillard‐derived volatile release and starch matrix dissolution, creating more complex and multidimensional sensory profiles (Pramudya and Seo 2018; Tobías‐Espinoza et al. 2024). However, the underlying physicochemical mechanisms were not directly examined and should therefore be interpreted cautiously.

**FIGURE 4:**
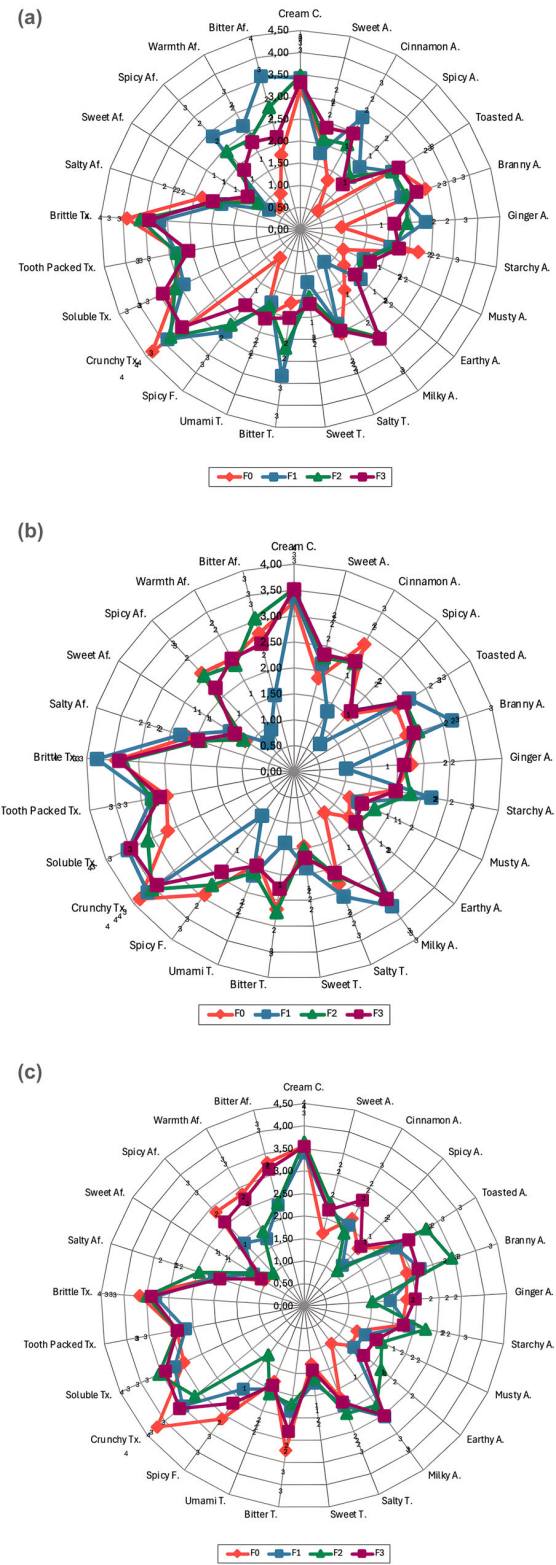
Radar plot with average ratings of RATA sensory attributes (on scale 1 to 5. with 1 = *low intensity* to 5 = *strongly intensity* on different condition serving (a) non milk serving (b) cold milk serving (c) warm milk serving for Control = formula with 0% SB; *F*1 = formula with 1% SB; *F*2 = formula with 2% SB; *F*3 = formula with 3% SB; SB = Spices blend.

The radar plots in Figure 4 further illustrated the effect of serving condition on selected RATA attributes. Ginger aroma, warming aftertaste, and soluble texture showed pronounced changes among serving conditions. For example, the mean intensity of warming aftertaste decreased from 2.89 in the dry condition to 0.76 with cold milk and subsequently increased to 1.93 with warm milk condition. This pattern suggests that cold milk suppressed the perceived warming sensation, whereas warm milk partially restored it, highlighting previous hypothesis for temperature‐dependent trigeminal perception. The response may reflect temperature‐dependent changes in trigeminal perception and the release or dilution of pungent compounds; however, receptor‐level responses were not measured.

Soluble texture also changed with serving condition. For example, its mean intensity for control *F*0 decreased from 4.31 in the dry condition to 3.49 with cold milk. This decrease reflects the effect of milk contact on perceived cereal breakdown and hydration, although the values should be interpreted as consumer‐rated intensities rather than direct measurements of hydration kinetics (Dias‐Faceto and Conti‐Silva 2022).

The radar profiles of *F*2 and *F*3 became more similar under warm‐milk condition, consistent with their reduced separation in the PCA map. Together, the PCA and radar‐plot results demonstrate that the sensory distinctions among formulations depended not only on spice‐blend level but also on the serving condition. These findings support evaluating breakfast cereals under realistic consumption conditions because the sensory profile observed for the dry product may not fully represent its perception when consumed with milk (Borgognone et al. 2001).

#### 3.3.3 Hedonic Evaluation

**FIGURE 5:**
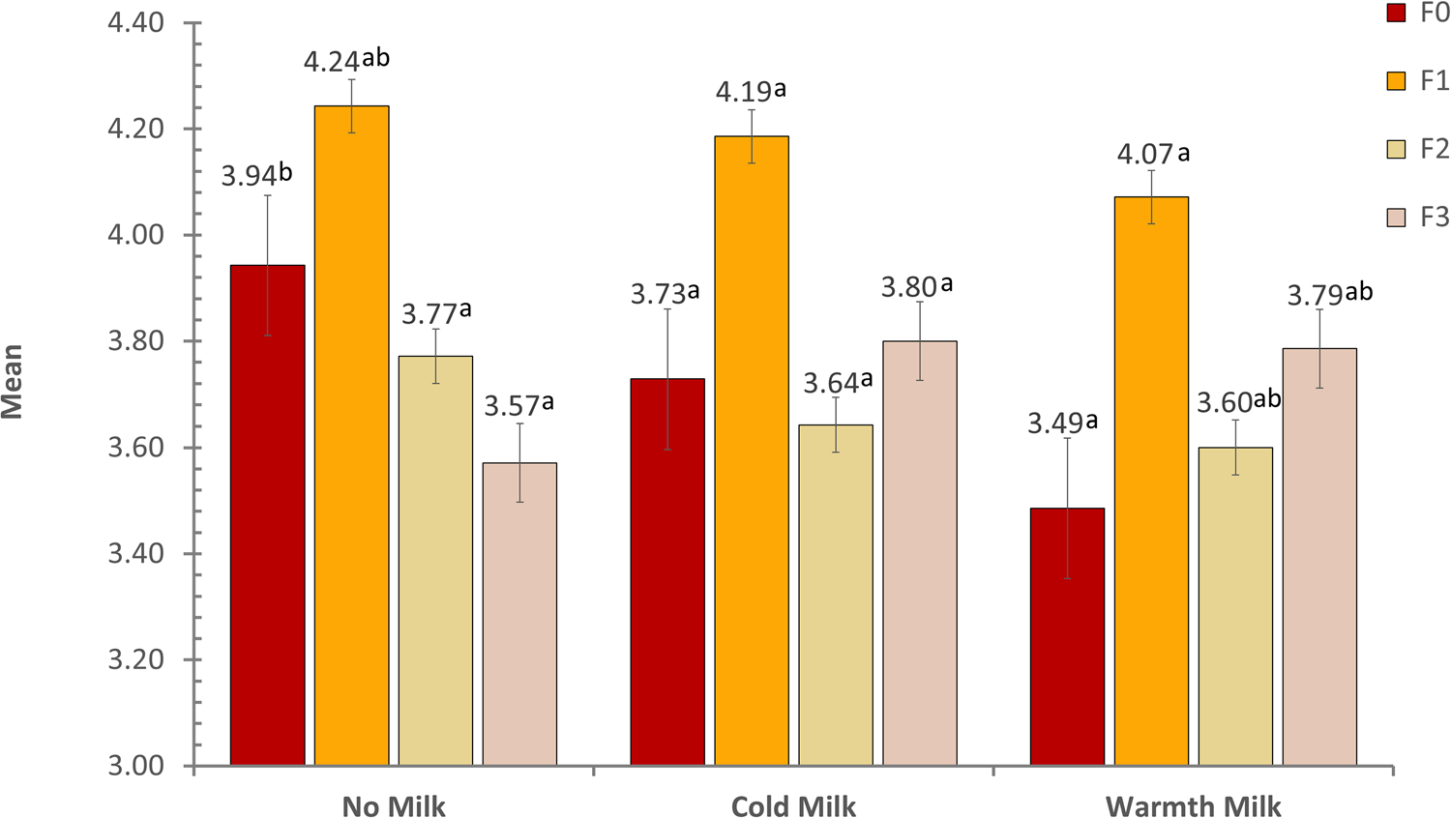
Overall rating hedonic spice‐enriched extruded breakfast cereal on different condition serving. Error bars = ±SE (pooled MSE from ANOVA = 1.326. *n* = 70). Different letters indicate significant differences (*p* < 0.05. Duncan's test).

Hedonic evaluation showed that consumer acceptance depended on serving conditions (Figure 5), supporting the RATA data. Under the dry condition, the *F*1 formula obtained the highest mean liking score of 4.24. This score was significantly higher than those of *F*2 and *F*3 with 2% and 3% spice‐blend incorporation, with received mean scores of 3.77 and 3.57, respectively, whereas the control *F*0 was statistically comparable to the *F*1, with mean score of 3.94. These findings indicate that the addition of 1% spice‐blend maintained or moderately improved consumer acceptance, whereas higher spice‐blend concentrations reduced liking, possibly because of the greater perception of pungent, bitter, and persistent spice‐related attributes identified through the CATA, RATA, and penalty analyses.

When the cereals were served with cold milk, no significant differences in overall liking were observed among the formulations (Figure 5). This result indicates that the sensory differences caused by spice‐blend concentration had a comparatively limited effect on overall acceptance under the cold‐milk condition. The milk matrix may have reduced perceptual contrasts among the formulations through dilution, flavor masking, or altered release of aroma and taste compounds; however, these mechanisms were not directly measured in the present study (Dias‐Faceto and Conti‐Silva 2022). The convergence of scores under cold milk also reflects alignment with the common conventional cold breakfast cereal consumption context, spice volatiles suppressed for all formulations fell within the sensory expectations of conventional cold breakfast cereal, leaving little basis for discrimination even *F*1 held the numerically highest score acceptance.

Under the warm milk condition, significant differences among formulations were observed again, with the *F*1 formula achieving the highest acceptance with mean liking score of 4.07. The favorable acceptance of this formulation was consistent with its association with sweet aroma, milky aroma, and sweet taste in the RATA analysis, together with a comparatively moderate perception of bitter and pungent attributes. Warm milk may have modified the release and perception of spice‐derived volatile compounds, thereby increasing differentiation among the formulations. At the *F*1, the spice blend may have contributed additional aroma complexity (reflects the thermal enhancement of spice‐derived volatiles release) without producing the excessive bitterness, pungency, or persistent aftertaste associated with the higher spice‐blend concentrations (Proserpio et al. 2020; Amer and Rizk 2022). Nevertheless, the individual volatile compounds responsible for these effects were not quantified in the present study.

Thus, this finding demonstrate that 1% SB represent the optimal balance between sensory enhancement and consumer acceptance, particularly under realistic consumption conditions involving milk. The results further highlight the importance of serving context in determining consumer perception and preference of extruded cereal products.

#### 3.3.4 Integrated Sensory Analysis Interpretation

The combined hedonic, CATA, RATA, and penalty‐analysis results indicate that the 1% spice‐blend formulation (*F*1) provided the most favorable balance between spice‐related sensory characteristics and consumer acceptance. However, because the liking score of the *F*1 formula did not differ significantly from that of the control (*F*0) under the dry condition and no formulation differences were detected under the cold‐milk condition, it is more appropriate to describe *F*1 as the most consistently accepted spice‐enriched formulation rather than as universally “superior” formula under every serving condition. These findings also demonstrate that serving context substantially influenced product perception and should therefore be considered when developing and evaluating extruded breakfast cereals intended to be consumed with milk.

### 3.4 Determination of the Best Formulation

The best‐performing formulation was selected using Bayesian multi‐criteria decision analysis, which integrated physicochemical and sensory attributes into a single weighted score for each formulation. Nine predefined criteria were included: color, hardness, crunchiness or crispness, cereal resistance in milk, WAI, WSI, BD, SE, and overall hedonic liking (Table 8). The weighting factors were assigned a priori according to the product‐development objectives, with overall hedonic liking receiving the highest priority because consumer acceptance was considered essential for formulation selection.

**TABLE 8:** Selection of the best formula of spice‐enriched extruded breakfast cereal based on the Bayesian method.

Formula	Criterion									Alternative value	Rank
	*A*	*B*	*C*	*D*	*E*	*F*	*G*	*H*	*I*		
*F*0	1	1	2	2	4	2	4	3	3	2.46	4
*F*1	2	2	3	3	3	4	2	2	4	2.84	1
*F*2	3	4	1	4	2	3	3	1	1	2.37	3
*F*3	4	3	4	1	1	1	1	4	2	2.33	2
Criterion weight	0.09	0.10	0.15	0.13	0.07	0.08	0.13	0.08	0.16		

*Note*: Control = formula with 0% SB—Control; *F*1 = formula with 1% SB; *F*2 = formula with 2% SB; *F*3 = formula with 3% SB; *A* = color; *B* = hardness texture; *C* = crunchiness texture; *D* = resistance of cereal in milk; *E* = water absorption index; *F* = water solubility index; *G* = bulk density; *H* = sectional expansion; *I* = overall liking rating hedonic.

Abbreviation: SB, Spices blend.

The *F*1 achieved the highest overall score (Table 8) and was therefore selected for further specific functional characterization in present study; that is GI analysis. This formulation performed the most favorable balance among the evaluated criteria rather than the best value for every individual parameter. Its expansion and textural properties remained generally comparable to those of the control *F*0, while it obtained the highest mean hedonic score under both the dry and warm‐milk serving conditions. In contrast, the *F*2 and *F*3 received lower overall scores, primarily because their lower hedonic ratings outweighed improvements in some physicochemical or functional properties. The reduced acceptance of these formulations was consistent with the stronger perception of bitter, pungent, and persistent spice‐related attributes identified through the CATA, RATA, and penalty analyses.

The control *F*0 showed comparable performance for several physical and textural criteria with *F*1 (1% spice‐blend incorporation) but lacked the aromatic and functional contribution of the spice blend. Accordingly, the *F*1 was selected as the most appropriate compromise between product physical performance, spice incorporation, and consumer acceptance within the predefined decision framework. The selected best formulation was subsequently analyzed for TDF and GI.

Beyond the individual effects of spice incorporation, the principal contribution of this study lies in integrating consumer perception, physicochemical performance, and functional attributes within a unified product‐development framework. No prior study on extruded breakfast cereals has combined a consumer‐oriented formulation strategy, Bayesian multi‐criteria decision analysis, multi‐method sensory profiling (CATA, RATA, hedonic evaluation), physicochemical characterization, and GI assessment in a single framework. The combined use of CATA, RATA, hedonic and penalty analyses provided complementary information on sensory drivers and barriers to acceptance, while Bayesian multi‐criteria decision analysis enabled these consumer responses to be considered alongside physicochemical characteristics in formulation selection. Earlier work on spice‐ or bran‐enriched extrudates has generally treated sensory acceptability and functional performance as distinct aspects. Subsequent antioxidant and glycemic assessments further established the functional performance of the selected product. In this study, the integrated framework enables the identification of formulation candidates from both consumer‐oriented and functionality‐oriented perspectives, followed by their integration to determine the overall optimal formulation. This integrated approach therefore extends conventional formulation studies by demonstrating that functional enhancement should be optimized concurrently with technological quality and consumer acceptance rather than evaluated as independent outcomes.

### 3.5 TPC and TFC

TPC increased significantly with spice‐blend concentration (*p* < 0.001), from 17.53 to 29.16 mg GAE/g (Table 9). This increase was primarily associated with the contribution of spice‐derived phenolics, including cinnamic acid and hydroxycinnamic acid derivatives (Przygodzka et al. 2015). Extrusion may also have increased the extractability of bound phenolics through thermal and mechanical disruption of the cereal matrix (by releasing it from cereal matrix); however, phenolic retention or release by moderate extrusion temperatures (120°C–145°C) was not directly evaluated against the unprocessed mixture (Delgado‐Licon et al. 2009; Leyva‐Corral et al. 2016).

**TABLE 9:** Bioactive properties of spice‐enriched extruded breakfast cereal.

Bioactive parameter	Formula			
	*F*0	*F*1	*F*2	*F*3
DPPH (mg TE/g)	4.2528 ± 0.0042^d^	15.4716 ± 0.0019^c^	29.6970 ± 0.0023^b^	30.7430 ± 0.0020^a^
ABTS(mg TE/g)	6.0827 ± 0.0126^d^	46.8703 ± 0.0043^c^	82.5036 ± 0.0184^b^	103.3599 ± 0.0101^a^
TPC (mg GAE/g)	17.5316 ± 0.0021^d^	19.7107 ± 0.0017^c^	27.02 ± 0.0005^b^	29.16 ± 0.0011^a^
TFC (mg QE/g)	9.5064 ± 0.0721^c^	12.6029 ± 0.0012^b^	22.2531 ± 0.0022^b^	26.9576 ± 0.0037^a^

*Note*: Mean ± standard deviation values in each column with the different letters are significantly different (*p* ≤ 0.05). *F*0 = formula with 0% SB—Control; *F*1 = formula with 1% SB; *F*2 = formula with 2% SB; *F*3 = formula with 3% SB.

Abbreviation: SB, Spices blend.

TFC similarly increased from 9.51 to 26.96 mg QE/g, although the *F*1 and *F*2 did not differ significantly (*p* = 0.105) (Table 9). TFC remained lower than TPC might be attributed to both analytical selectivity and compositional differences of the spice‐blend. The AlCl_3_ method selectively measures certain flavonoid structures (flavones and flavanols containing specific functional groups (C‐4 keto and C‐3/C‐5 hydroxyl groups)), whereas the Folin–Ciocalteu assay also responds to non‐flavonoid phenolics (Panche et al. 2016; Munteanu and Apetrei 2021). The spice blend contained both non‐flavonoid phenolics, such as gingerols, shogaols, and cinnamic derivatives, and flavonoids derived mainly from lemongrass and ginger (Khasanah et al. 2024; Sari et al. 2026). Consistent with this compositional shift, the ATR‐FTIR spectra (Section 3.7) showed increased intensity at the 1750 and 1240 cm^−1^ bands, assigned to carbonyl and phenolic C─O stretching, which correspond to the elevated phenolic and flavonoid contents in the spice‐enriched formulations.

### 3.6 Antioxidant Activity (ABTS and DPPH Assay)

DPPH and ABTS antioxidant activities increased significantly with spice‐blend concentration (*p* < 0.001) (Table 9). DPPH values increased from 4.25 to 30.74 mg TE/g, whereas ABTS values increased from 6.08 to 103.36 mg TE/g. The stronger ABTS response likely reflects its ability to detect a broader range of hydrophilic and lipophilic antioxidants, while DPPH is generally more selective toward compounds capable of donating hydrogen atoms or electrons in an organic medium (Floegel et al. 2011; Angeli et al. 2021). As a results, the ABTS assay likely captured a broader spectrum of antioxidant compounds, whereas DPPH predominantly reflected the phenolic fraction activity (Kiss et al. 2025; Sari et al. 2026).

Flavonoids such as apigenin and luteolin derivatives from lemongrass and quercetin glycosides from ginger, which possess multiple hydroxyl groups and conjugated B‐ring structures, are known to exhibit stronger electron transfer activity, further explaining the higher ABTS response (M. Wang et al. 2019). ABTS was better positioned to capture this chemically heterogenous pool, while DPPH predominantly reflected the phenolic fraction (Abramovič et al. 2018). The absence of significant difference in protein and moisture contents among formulations (*F*0–*F*3) further supported that the observed antioxidant activity is primarily driven by non‐peptide bioactive compounds originating from the spice blend rather than protein‐derived peptides or extrusion process artifacts (Arribas et al. 2019). These results implied that the ABTS assay provides a more comprehensive estimation of antioxidant capacity in this multi‐component cereal system, whereas DPPH assay offers a complementary but more selective assessment of phenolic‐centric antioxidant activity (Abramovič et al. 2018).

The increase in antioxidant activity in this study was consistent with the higher TPC and TFC values (Table 9) and the contribution of spice‐derived compounds, including gingerols, shogaols, cinnamic derivatives, citral, and flavonoids. Extrusion may have modified their extractability through matrix disruption, although compound‐specific retention and degradation were not measured (Qiao et al. 2021; Kumar and Singh 2024). In addition, synergistic interactions among antioxidants from multiple sources (including spice‐derived phenolics, tocopherols, and γ‐oryzanol from rice bran, and phenolic compounds associated with resistant starch in unripe banana flour) may contribute to the enhanced antioxidant capacity, particularly as detected by ABTS (Limsangouan et al. 2010; Sarawong et al. 2014; P. Wang et al. 2018). Thus, ABTS and DPPH provided complementary estimates of antioxidant capacity in the multicomponent cereal matrix.

The ATR‐FTIR spectra (Section 3.7) point the same way. The O─H stretching band (∼3300 cm^−1^) and the C─H stretching bands (2925 and 2852 cm^−1^) grew stronger with increasing spice levels consistent with an accumulation of hydroxyl‐ and aliphatic‐chain‐bearing compounds behind the antioxidant response.

### 3.7 ATR‐FTIR Fingerprinting

**FIGURE 6:**
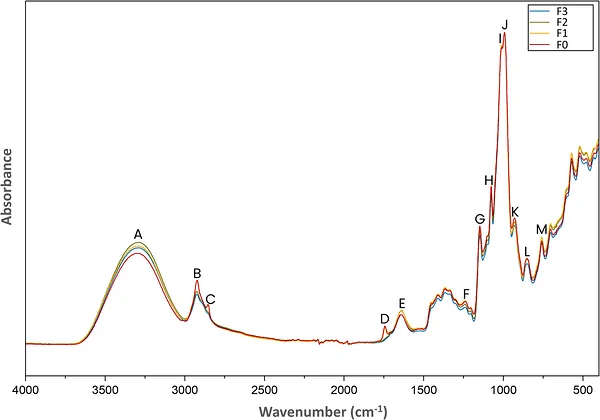
ATR‐FTIR spectra of SEBC scanned at wavenumbers of 4000–400 cm^−1^. Peak A = 3300 cm^−1^ hydrogen‐bonded O─H stretching vibration; Peak B = 2925 cm^−1^ asymmetric CH_3_ stretching; Peak C = 2852 cm^−1^ symmetric C─H stretching of CH_2_; Peak D = 1750 cm^−1^ C═O stretching of carbonyl groups; Peak E = 1640 cm^−1^ C═C stretching and N─H bending; Peak F = 1240 cm^−1^ phenolic C─O stretching; Peak G = 1150 cm^−1^ C─O bonds or O─H deformation; Peak H = 1057 cm^−1^ polysaccharide C─O─C stretching; Peak I = 1014 cm^−1^ C─O stretching and C─O─C/C─O bending; Peak J = 993 cm^−1^ C═O stretching of the glucose ring; Peak K = 930 cm^−1^ C─H deformation at the anomeric carbon of α‐glucose; Peak L = 851 cm^−1^ C─O─C of native starch linkages; Peak M = 760 cm^−1^ conjugated aromatic C─H out‐of‐plane bending.

FTIR‐ATR fingerprinting was used supporting characterization to evaluate formulation‐related differences at the functional‐group level. Spectra from 4000–400 cm^−1^ were broadly similar across formulations *F*0–*F*3, reflecting the starch‐dominant cereal matrix, while changes in peak or band intensity and broadening increased with spice‐blend concentration (Figure 6). Each spectrum obtained functioning as a chemical fingerprint of formulation‐level composition in the present study (Rohman et al. 2011).

The broad band near 3300 cm^−1^ was assigned to hydrogen‐bonded O─H stretching, combining the signal from starch hydroxyl groups and phenolic compounds introduced by the spice blend, this trend reflecting a higher density of hydroxyl groups from starch and spice‐derived phenolics available for water binding consistent with the higher WAI observed in *F*1–*F*3. The bands at 2925 and 2852 cm^−1^ represented aliphatic C─H stretching (including asymmetric CH_3_ and symmetric CH_2_ stretching), intensifying at higher spice concentrations in line with the aliphatic chain contribution of essential oil constituents. The bands near 1750 and 1240 cm^−1^ were associated with carbonyl C═O stretch and phenolic C─O groups stretch, respectively, grew more pronounced with spice addition. Meanwhile, the regions at 1057 cm^−1^ with characteristics of polysaccharide C─O─C region and 930–851 cm^−1^ with characteristic of glycosidic bond signatures remained consistent across formulations, reflecting the starch‐dominant base matrix. The non‐linear hardness and crispness responses more likely reflect differences in matrix porosity, expansion, and density than any chemical change in the starch polymer. These spectral differences are consistent with the contribution of spice‐derived constituents; however, they do not confirm specific molecular interactions or individual compounds.

In this study, the relatively wide variation in antioxidant activity, TPC, and TFC values across formulations is advantageous for multivariate analysis, as it provides a calibrated and predictive model (Kusumadewi et al. 2022). The effect of spice addition on functional group composition indicates that spice components introduce bioactive constituents distinct from those of the base matrix. Further chemometric analysis and multivariate analysis is required to systematically quantify these differences.

The variation among spectra supported subsequent chemometric discrimination of the formulations *F*0–*F*3 in the present study. The ATR‐FTIR fingerprinting confirmed the presence of phenolic and flavonoid compounds in SEBC. These compounds are known to dominantly contribute to the spice‐derived antioxidant activity. Nevertheless, ATR‐FTIR should be considered a rapid compositional fingerprinting approach rather than direct evidence of phenolic concentration, antioxidant activity, or starch–polyphenol complex formation. Overall, the FTIR fingerprint provides structural confirmation of the TPC, TFC, and antioxidant trends, reinforcing that the spice‐induced increase in bioactivity is accompanied by corresponding molecular modifications in the cereal matrix

### 3.8 Glycemic Index

The *F*1 containing 1% spice‐blend was selected as best formula based on its overall balance of physicochemical properties and consumer acceptance, despite no significant differences in TDF among formulations *F*0–*F*3. GI assessment of this formulation, conducted according to ISO 26642:2010, yielded a value of 47 ± 8 (glucose = 100), classifying the product as low‐GI food (GI < 55). This low glycemic response might be associated with the combine effects of formulation composition and structural modification induced by extrusion.

The low GI may reflect the combined effects of rice bran, unripe banana flour, and the spice‐incorporated product matrix. Dietary fiber and resistant starch from these ingredients can reduce starch accessibility and slow glucose release during digestion, by modulate glucose absorption and delay gastric emptying (Kumar et al. 2022; Khoza et al. 2023; Dibakoane et al. 2024). Kumar et al. (2022) demonstrated that higher resistant starch content significantly correlated with lower GI values of rice varieties. Unripe banana flour contributes resistant starch, which reduces starch digestibility by limiting enzyme accessibility through its compact granular structure and higher amylose content (Dibakoane et al. 2024). Khoza et al. (2023) found that green banana flour able to reduced GI and lower risk of type 2 diabetes and obesity, with resistant starch content remaining high even after thermal processing. These structural characteristics slow glucose release during digestion, contributing to a reduced postprandial glycemic response.

Spice‐derived bioactive compounds, particularly those from cinnamon, may also influence carbohydrate digestion through inhibition of enzymes such as α‐glucosidase (Moreira et al. 2024). Cinnamon, in particular, has been widely reported to inhibit carbohydrate‐hydrolyzing enzymes such as α‐glucosidase, thereby reducing glucose release during digestion (resulting hypoglycemic properties). Clinical evidence indicates that cinnamon supplementation can attenuate postprandial hyperglycemia through mechanisms comparable to α‐glucosidase inhibitors. In the current study, ginger and lemongrass‐derived bioactives may also contributed through antioxidant activity and metabolic pathways, although their effects are less pronounced. However, resistant‐starch content, enzyme inhibition, and starch–polyphenol interactions were not directly measured in this study; further studies are needed to confirm the mechanism.

The low GI was achieved despite the product's relatively high carbohydrate content, indicating that carbohydrate quality and matrix structure may be more relevant than total carbohydrate content alone in determining glycemic response. The extrusion process may further enhance this effect by modifying starch structure and promoting interactions between starch, fiber, and polyphenols, which collectively reduce enzymatic digestibility. Given its low GI classification, the *F*1 formula combined acceptable sensory properties with a low glycemic response, supporting its potential as a functional breakfast cereal.

From an industrial perspective, the optimized 1% SB formulation is particularly relevant because functional enhancement was achieved through a relatively low level of spice incorporation without compromising consumer acceptance or requiring post‐extrusion flavor correction. The processing conditions used here sit within the standard operating range for commercial twin‐screw extrusion, so the *F*1 formulation should scale to industrial production without major process changes. Its low GI and favorable sensory scores make it a plausible option for manufacturers developing cereals aimed at glycemic management. The formulation uses ingredients compatible with conventional dry blending and extrusion operations, supporting its potential adaptation to existing cereal‐processing lines. Moreover, the use of rice bran and unripe banana flour provides opportunities for value addition to underutilized agricultural resources. Nevertheless, pilot‐ and industrial‐scale validation is required to confirm process stability, product consistency, and economic feasibility before commercial application.

## 4 Conclusion

The formulation containing 1% spice‐blend (*F*1) was selected as the best formula through Bayesian multi‐criteria decision analysis integrating physicochemical and sensory attributes. The control *F*0 showed acceptable sensory quality with limited functional enhancement, whereas 3% spice‐blend increased antioxidant capacity but reduced liking because of stronger bitterness and pungency. These findings emphasize the need to balance functional improvement with sensory acceptance.

At *F*1 with 1% spice‐blend concentration, the cereal maintained favorable structural properties and achieved most consistent consumer acceptance across serving conditions. Serving conditions influenced perception: dry consumption resulted greater differentiation among formulations, cold fresh milk reduced sensory contrast, and warm fresh milk modified the perception of spice‐related attributes. Sweet taste was the main positive driver of liking, whereas bitter aftertaste was the principal negative attributes.

Spice incorporation increased phenolic content, flavonoid content, and antioxidant activity. The best formulation *F*1 also exhibited a low GI of 47 ± 8. However, the mechanisms underlying antioxidant retention, matrix interactions, and glycemic response were not directly verified in this study and require further structural and compound‐specific investigation. While our findings are promising, they are constrained by the absence of shelf‐life evaluation, the limited demographic scope of the sensory panel, and the lack of in vivo glycemic testing. Addressing these aspects in future research would further strengthen the product's commercial and clinical relevance. This study offers a practical approach for developing consumer‐acceptable extruded breakfast cereals with enhanced functional properties by moderate spice‐blend incorporation at 1%.

## Author Contributions

**Zaki Muhammad Husyemi Rafsanjani**: investigation, writing – original draft, methodology, formal analysis, data curation, conceptualization, visualization, project administration, funding acquisition. **Slamet Budijanto**: conceptualization, supervision, writing – review and editing, resources, funding acquisition. **Vallerina Armetha**: conceptualization, methodology, writing – original draft, writing – review and editing, resources, funding acquisition, supervision, validation. **Azis Boing Sitanggang**: conceptualization, supervision, writing – review and editing, methodology, resources.

## Conflicts of Interest

The authors declare no conflicts of interest.
